# Acute ileitis facilitates infection with multidrug resistant *Pseudomonas aeruginosa* in human microbiota-associated mice

**DOI:** 10.1186/s13099-017-0154-4

**Published:** 2017-01-18

**Authors:** Eliane von Klitzing, Ira Ekmekciu, Stefan Bereswill, Markus M. Heimesaat

**Affiliations:** Department of Microbiology and Hygiene, Charité-University Medicine Berlin, CC5, Campus Benjamin Franklin, FEM, Garystr. 5, 14195 Berlin, Germany

**Keywords:** *Pseudomonas aeruginosa*, Multidrug resistant Gram-negative bacteria, Susceptibility to infection, Intestinal microbiota, Fecal transplantation, Human microbiota-associated mice, *Toxoplasma gondii* induced acute ileitis, Pro-inflammatory immune responses, Extra-intestinal and systemic sequelae of infection, Bacterial translocation

## Abstract

**Background:**

The rising incidence of multidrug resistant (MDR) Gram-negative bacteria including *Pseudomonas aeruginosa* has become a serious issue in prevention of its spread particularly among hospitalized patients. It is, however, unclear whether distinct conditions such as acute intestinal inflammation facilitate *P. aeruginosa* infection of vertebrate hosts.

**Methods and results:**

To address this, we analysed *P. aeruginosa* infection in human microbiota-associated (hma) mice with acute ileitis induced by peroral *Toxoplasma gondii* challenge. When perorally infected with *P. aeruginosa* at day 3 post ileitis induction, hma mice displayed higher intestinal *P. aeruginosa* loads as compared to hma mice without ileitis. However, the overall intestinal microbiota composition was not disturbed by *P. aeruginosa* (except for lowered bifidobacterial populations), and the infection did not further enhance ileal immune cell responses. Pro-inflammatory cytokines including IFN-γ and IL-12p70 were similarly increased in ileum and mesenteric lymph nodes of *P. aeruginosa* infected and uninfected hma mice with ileitis. The anti-inflammatory cytokine IL-10 increased multifold upon ileitis induction, but interestingly more distinctly in *P. aeruginosa* infected as compared to uninfected controls. Immune responses were not restricted to the intestines as indicated by elevated pro-inflammatory cytokine levels in liver and kidney upon ileitis induction. However, except for hepatic TNF-α levels, *P. aeruginosa* infection did not result in more distinct pro-inflammatory cytokine secretion in liver and kidney of hma mice with ileitis. Whereas viable intestinal bacteria were more frequently detected in systemic compartments such as spleen and cardiac blood *of P. aeruginosa* infected than uninfected mice at day 7 following ileitis induction, *P. aeruginosa* infection did not exacerbate systemic pro-inflammatory sequelae, but resulted in lower IL-10 serum levels.

**Conclusion:**

Acute intestinal inflammation facilitates infection of the vertebrate host with MDR bacteria including *P. aeruginosa* and might also pose particularly hospitalized patients at risk for acquisition. Since acute *T. gondii* induced inflammation might mask immunopathology caused by *P. aeruginosa*, a subacute or chronic inflammation model might be better suited to investigate the potential role of *P. aeruginosa* infection in the aggravation of intestinal disease.

**Electronic supplementary material:**

The online version of this article (doi:10.1186/s13099-017-0154-4) contains supplementary material, which is available to authorized users.

## Background

*Pseudomonas aeruginosa*, a non-fermenting Gram-negative rod, is one of the most important bacterial pathogens responsible for a multitude of opportunistic infections in humans, especially in hospitalized patients [1]. The strictly aerobic pathogen is the primary cause of ventilator-associated pneumonia or superinfection of burn wounds associated with a mortality of more than 30% [2]. Its worldwide increasing resistance to manifold antibiotic classes particularly due to extended-spectrum β-lactamases, carbapenemases and 16S rRNA methylases makes *P. aeruginosa* to a continuously growing threat for immune-compromised individuals, patients suffering from cystic fibrosis and other pulmonary diseases or patients admitted to intensive care units [2–4]. A single flagellum and multiple cell surface pili allow motility and adherence of *P. aeruginosa* with surfaces [5], whereas biofilm formation, alginate secretion, quorum-sensing and an elaborated secretion system contribute to the virulence of *P. aeruginosa* [1, 5]. Earlier studies revealed that ingested *P. aeruginosa* are detectable in human fecal samples of healthy volunteers up to 6 days following oral administration without occurrence of clinical symptoms. Fecal *P. aeruginosa* loads were lower than those that had been ingested and decreased over time post challenge [6]. However, admission to a surgical ward may increase the risk of peroral *P. aeruginosa* acquisition, since the percentage of patients’ stool samples carrying *P. aeruginosa* increased during hospital stay [7]. In addition to objects and materials present on wards, the human gastrointestinal tract may be an important source of *P. aeruginosa* infection [7]. Recent clinical surveys revealed that *P. aeruginosa* detection rates were significantly higher in fecal and mucosal samples derived from patients suffering from irritable bowel syndrome [8] or in the colonic mucosa of a pediatric patient with ulcerative colitis [9] as compared to a healthy individual. Whereas the pathogenic potential of *P. aeruginosa* is well-known, its potential contribution to initiation and perpetuation of intestinal immunopathological conditions are not yet understood. Hence, there is currently a large gap in knowledge regarding the interplay of *P. aeruginosa*, the host microbiota and immune system, particularly under conditions of intestinal inflammation.

Within 1 week following peroral high dose infection with the intracellular parasite *Toxoplasma gondii*, susceptible mice develop acute inflammation of the terminal ileum with massive necrosis (pan-ileitis) as well as extra-intestinal and systemic sequelae and succumb to infection within 7–10 days [10–12]. This fatal hyper-inflammatory scenario is due to a typical T helper cell 1 (Th1)-type immunopathology and characterized by an overproduction of pro-inflammatory mediators including interferon (IFN)-γ, tumor necrosis factor (TNF)-α, nitric oxide, interleukin (IL)-6, IL-12 and monocyte chemoattractant protein (MCP)-1 evolving upon parasitic interacting with antigen presenting cells with subsequent activation of CD4+ T cells, whereas the *T. gondii* induced anti-inflammatory measures include IL-10 expression (reviewed by [13]). We showed earlier that the gut microbiota is essential for the initiation and progression of *T. gondii* induced ileitis and that small intestinal inflammation is accompanied by distinct changes in the commensal microbiota composition with Gram-negative species such as enterobacteria and *Bacteroides/Prevotella* spp. overgrowing the inflamed ileal lumen [11]. Toll-like receptor (TLR)-4 dependent signaling of lipopolysaccharide derived from Gram-negative intestinal commensals further perpetuates the hyper-inflammatory process [14, 15]. Hence, the Th1-type immunopathology underlying *T. gondii* induced ileitis and associated intestinal microbiota shifts resemble key features of acute episodes in patients suffering from Crohn’s disease [10, 13]. In the present study we investigated whether acute small intestinal inflammation predisposes the vertebrate host with a human microbiota to infection with multidrug resistant (MDR) *P. aeruginosa*. To address this, mice harboring a complex human microbiota were challenged with high dose *T. gondii* infection for ileitis induction and subsequently infected with a MDR *P. aeruginosa* strain. The presented results shed further light onto the interplay between MDR *P. aeruginosa*, the host innate and adaptive immunity and human intestinal microbiota during acute small intestinal inflammation.

## Methods

### Ethical statement

All animal experiments were conducted according to the European Guidelines for animal welfare (2010/63/EU) with approval of the commission for animal experiments headed by the “Landesamt für Gesundheit und Soziales” (LaGeSo, Berlin; registration numbers G0097/12 and G0039/15). Animal welfare was monitored twice daily by assessment of clinical conditions and weight loss of mice. Mice suffering from weight loss >20% were euthanized by isoflurane treatment (Abbott, Germany) in accordance with the guidelines of the local authorities.

### Generation of gnotobiotic (secondary abiotic) mice

Female C57BL/6j mice were bred under specific pathogen-free conditions in the Forschungsinstitute für Experimentelle Medizin (Charité-University Medicine, Berlin, Germany). Gnotobiotic (i.e. secondary abiotic) mice with a virtually depleted microbiota were generated as described previously [11]. In brief, 8 weeks old mice were transferred to sterile cages and subjected to a broad-spectrum antibiotic treatment for 8–10 weeks by adding ampicillin plus sulbactam (1 g/L; Ratiopharm, Germany), vancomycin (500 mg/L; Cell Pharm, Germany), ciprofloxacin (200 mg/L; Bayer Vital, Germany), imipenem (250 mg/L; MSD, Germany) and metronidazole (1 g/L; Fresenius, Germany) to the drinking water (ad libitum).

### Generation of human microbiota-associated mice

Fresh fecal samples free of enteropathogenic bacteria, viruses and parasites were collected from five individual healthy volunteers, dissolved in sterile phosphate buffered saline (PBS; Gibco, life technologies, UK), aliquoted and stored at −80 °C as described earlier [16]. Immediately before reconstitution experiments, individual fecal aliquots were thawed, pooled, and the main bacterial communities within the donor suspension quantitatively assessed by cultural and molecular methods [16]. To generate human intestinal microbiota-associated (hma) mice, gnotobiotic animals were subjected to peroral fecal transplantations with 0.3 mL of the donor suspension by gavage on 3 consecutive days. The total load of bacterial groups between independent experiments counted around 10^10^ colony forming units (CFU) and varied less than 0.5 logarithmic orders of magnitude. To assure proper establishment of the human microbiota in the murine host, mice were kept for at least 3 weeks until ileitis induction. Immediately before peroral *T. gondii* infection individual fecal samples were collected for quantitative cultural and molecular analyses of main intestinal bacterial communities.

### Induction of acute ileitis

In order to induce acute ileitis mice harboring a human intestinal microbiota were infected perorally with 50 cysts of *T. gondii* (ME49 strain) by gavage as described previously [11, 14, 17].

### *Pseudomonas aeruginosa* infection

Three days following ileitis induction mice were perorally infected with 10^9^ CFU of a MDR *P. aeruginosa* strain by gavage in a total volume of 0.3 mL PBS. The *P. aeruginosa* isolate was cultured from respiratory material of a patient suffering from nosocomial pneumonia and kindly provided by Prof. Dr. Bastian Opitz (Charité-University Medicine, Berlin, Germany). Of note, the bacterial strain displayed antimicrobial sensitivity to fosfomycin and colistin only.

### Cultural analysis of the intestinal loads of *P. aeruginosa*

On days 2, 3 and 4 post *P. aeruginosa* infection individual fecal samples were homogenized in sterile PBS, and serial dilutions streaked onto Columbia agar supplemented with 5% sheep blood (Oxoid, Germany) and Cetrimid agar (Oxoid) and incubated in an aerobic atmosphere at 37 °C for at least 48 h to assess intestinal *P. aeruginosa* loads.

### Clinical conditions

Body weights as well as macroscopic and/or microscopic abundance of fecal blood were assessed in individual mice on a daily basis by the Guajac method using Haemoccult (Beckman Coulter/PCD, Germany).

### Sampling procedures

Mice were sacrificed 7 days after ileitis induction by isoflurane treatment (Abbott, Germany). Cardiac blood and tissue samples from spleen, liver, lung, kidney, mesenteric lymph nodes (MLN), ileum and colon were removed under sterile conditions. Ileal and colonic samples from each mouse were collected in parallel for microbiological, immunological, immunohistochemical and histopathological analyses. Experiments were repeated at least twice.

### Small intestinal lengths and histopathological scores

Small intestinal lengths were determined by measuring the distance from the duodenum leaving the stomach to the ileal-caecal transition. Ex vivo biopsies derived from the terminal ileum were immediately fixed in 5% formalin and embedded in paraffin. Sections (5 µm) were stained with hematoxylin and eosin (H&E) and subjected to a standardized histopathological scoring system ranging from 0 to 6 as described earlier [11, 14].

### Immunohistochemistry

5 µm thin paraffin sections of ileal ex vivo biopsies were used for in situ immunohistochemical analysis as described previously [18–20]. Primary antibodies against CD3 (Polycl.rabbit anti human, DAKO, Denmark; 1:10), FOXP3 (FJK-165, eBioscience, Germany; 1:100), B220 (eBioscience; 1:200) and F4/80 (biot. Clone BM 8 rat anti mouse, Life Technologies, USA; 1:100) were used to assess T lymphocytes, regulatory T cells (Treg), B lymphocytes and macrophages/monocytes, respectively. The average number of positively stained cells within at least six high power fields (HPF, 0.287 mm^2^; 400× magnification) were determined by an independent blinded investigator.

### Cultural survey of intestinal microbiota and bacterial translocation

For comprehensive quantitative survey of intestinal microbiota composition and translocation of viable bacteria to extra-intestinal compartments, colonic and ileal luminal contents as well as ex vivo biopsies derived from MLN, spleen, liver and lung, respectively, were homogenized in sterile PBS and analyzed in serial dilutions on different solid culture media as described earlier [11, 14, 21]. Cardiac blood was incubated in thioglycolate enrichment broths (BD Bioscience, Germany) for 1 week at 37 °C and streaked onto solid media thereafter. Bacteria were grown at 37 °C for at least 2–3 days under aerobic, microaerobic and anaerobic conditions.

### Molecular analysis of the ileal microbiota

DNA was extracted from fecal samples as described previously [11, 22]. In brief, DNA was quantified by using Quant-iT PicoGreen reagent (Invitrogen, UK) and adjusted to 1 ng per µL. Then, main bacterial groups abundant in the murine and human intestinal microbiota were assessed by quantitative real-time polymerase chain reaction (qRT-PCR) with species-, genera- or group-specific 16S rRNA gene primers (Tib MolBiol, Germany) as described previously [16, 18, 23] and numbers of 16S rRNA gene copies per ng DNA of each sample determined.

### Cytokine detection in colon, ileum, mesenteric lymph nodes, liver, kidney, spleen and serum

Ex vivo biopsies of approximately 1 cm^2^ (ileum cut longitudinally) were washed in PBS and placed in 24-flat-bottom well culture plates (Falcon, Germany) containing 500 mL serum-free RPMI 1640 medium (Gibco, life technologies) supplemented with penicillin (100 U/mL, Biochrom, Germany) and streptomycin (100 µg/mL; Biochrom). After 18 h at 37 °C, culture supernatants and serum samples were tested for IFN-γ, TNF-α, MCP, IL-12p10, IL-6 and IL-10 by the Mouse Inflammation Cytometric Bead Assay (CBA; BD Bioscience) on a BD FACSCanto II flow cytometer (BD Bioscience). Nitric oxide was determined by the Griess reaction as described previously [11].

### Statistical analysis

Mean values, medians, standard deviations (SD) and levels of significance were determined using appropriate tests as indicated (two-tailed Student’s t test, Mann–Whitney U test, ordinary one-way ANOVA and Kruskal–Wallis test). Two-sided probability (*p*) values ≤0.05 were considered significant.

## Results

### Acute ileitis induction facilitates multidrug resistant *P. aeruginosa* infection of human microbiota-associated mice

**Fig. 1:**
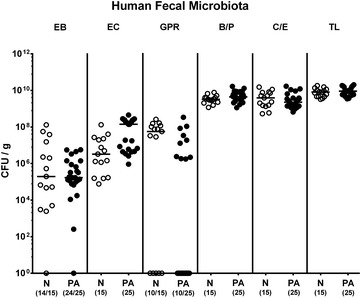
Intestinal microbiota composition following human fecal transplantations. Gnotobiotic (secondary abiotic) mice were generated by broad-spectrum antibiotic treatment and subjected to peroral transplantations of human feces as described in “Methods” section. Three weeks following association with human microbiota and immediately before acute ileitis induction, a cultural survey of the intestinal microbiota composition was performed on fecal samples derived from mice of the *P. aeruginosa* infection cohort (PA; *filled circles*) as compared to the non-infected control group (N; *open circles*). Numbers of viable enterobacteria (EB), enterococci (EC), Gram-positive rods (GPR), *Bacteroides/Prevotella* spp. (B/P), *Clostridium/Eubacterium* spp. (C/E) and the total loads (TL) were expressed as colony forming units per gram feces (CFU/g). Numbers of animals harboring the respective bacterial groups are given in parentheses and medians are indicated. Data shown were pooled from three independent experiments

The primary goal of our present study was to investigate the influence of acute intestinal inflammation on intestinal infection with MDR *P. aeruginosa* and its interplay with the intestinal commensal microbiota and the immune system in the vertebrate host. We first addressed whether acute ileitis impacted the intestinal colonization capacity of MDR *P. aeruginosa*. To accomplish this, we generated secondary abiotic mice with a virtually depleted microbiota by broad-spectrum antibiotic treatment and reconstituted these mice with a human intestinal microbiota by peroral transplantation of mixed fecal samples derived from five healthy individuals on 3 consecutive days. A comprehensive cultural survey of the main intestinal bacterial groups abundant in the human gut performed 3 weeks post fecal transplantation (and immediately before acute ileitis induction) revealed that the human microbiota had stably established within the murine host (Fig. 1). Furthermore, loads of respective bacterial groups did not differ between cohorts that were subsequently infected with MDR *P. aeruginosa* or remained uninfected (Fig. 1).

**Fig. 2:**
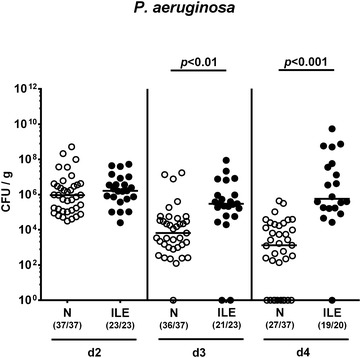
Kinetic of fecal multidrug resistant *P. aeruginosa* loads in human microbiota-associated mice with and without acute ileitis. Human microbiota-associated mice were perorally challenged with *T. gondii* ME49 to induce acute ileitis (ILE; *filled circles*) and additionally infected with MDR *P. aeruginosa* 3 days following ileitis induction. *P. aeruginosa* infected human microbiota-associated mice without ileitis induction (N; *open circles*) served as controls. Intestinal colonization densities were determined in fecal samples at days (d) 2, 3 and 4 following *P. aeruginosa* infection by culture and expressed as colony forming units per gram feces (CFU/g). Numbers of mice harboring *P. aeruginosa* out of the total number of analyzed mice are given in parentheses. Medians (*black bars*) and significance levels (*p* values) determined by Kruskal–Wallis test are indicated. Data shown were pooled from three independent experiments

In order to induce acute ileitis, hma mice were perorally infected with 50 cysts of *T. gondii* ME 49 strain by gavage. At day 3 following ileitis induction (a critical time point when initial histopathological changes within the ileal mucosa can be observed in peroral high dose *T. gondii* infection [11]), mice were perorally challenged with 10^9^ CFU *P. aeruginosa* by gavage. Naive hma mice served as uninfected controls. In addition, one separate cohort of hma mice without induced ileitis was included into the *P. aeruginosa* infection studies. Interestingly, mice with ileitis could be infected by *P. aeruginosa* and displayed median fecal pathogenic loads of approximately 10^6^ CFU per g that were two to three orders of magnitude higher as compared to those determined in hma mice without ileitis induction at days 3 and 4 post infection (p.i.) (p < 0.01 and p < 0.0001, respectively; Fig. 2). On day 4 p.i., 73.0% of hma mice without ileitis induction were carrying *P. aeruginosa*, whereas 95.0% of mice with an inflamed small intestine harbored the MDR bacteria (Fig. 2). Hence, acute small intestinal inflammation renders hma mice more susceptible to infection with MDR *P. aeruginosa*.

### Intestinal microbiota composition in multidrug resistant *P. aeruginosa* infected human microbiota-associated mice suffering from acute ileitis

**Fig. 3:**
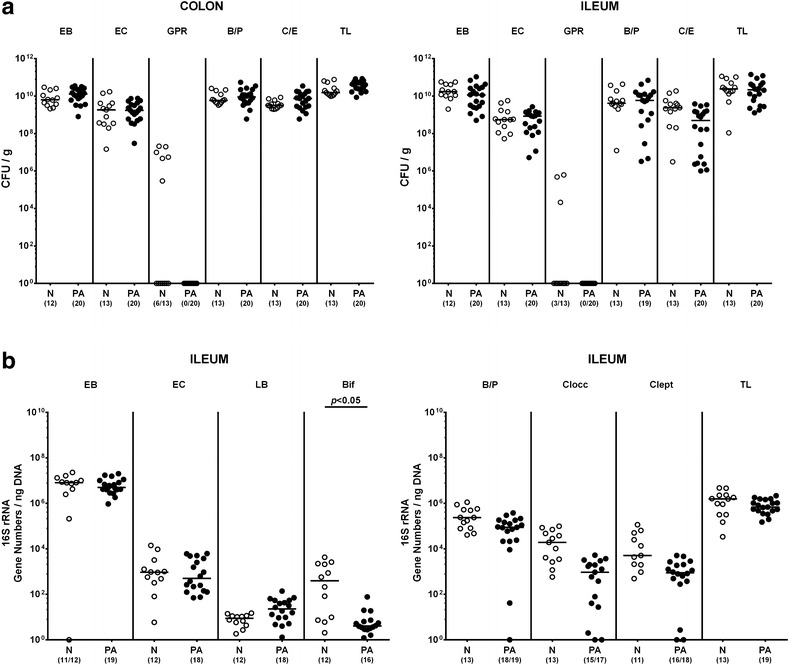
Intestinal microbiota analysis in multidrug resistant *P. aeruginosa* infected human microbiota-associated mice suffering from acute ileitis. Human microbiota-associated mice were perorally challenged with *T. gondii* ME49 to induce acute ileitis as described in "Methods" section. One group of mice (PA, *filled circles*) was additionally infected with MDR *P. aeruginosa* 3 days following ileitis induction by gavage, whereas non-infected mice served as controls with acute ileitis only (N, *open circles*). Main intestinal bacterial groups were quantified by culture and molecular analysis of luminal colon and ileum samples at day 7 post *T. gondii* infection. **a** Numbers of viable enterobacteria (EB), enterococci (EC), Gram-positive rods (GPR), *Bacteroides/Prevotella* spp. (B/P), *Clostridium/Eubacterium* spp. (C/E) and the total bacterial loads (TL) were determined in colonic and ileal luminal contents by culture and expressed as colony forming units per gram (CFU/g). **b** 16SrRNA of the main ileal bacterial groups including enterobacteria (EB), enterococci (EC), lactobacilli (LB), bifidobacteria (Bif), *Bacteroides/Prevotella* spp. (B/P), *Clostridium coccoides* group (Clocc), *Clostridium leptum* group (Clept) and total eubacterial loads (TL) are expressed as gene numbers per ng DNA. Numbers of animals harboring the respective bacterial group are given in parentheses. Medians and significance levels (*p* values) determined by Kruskal–Wallis test are indicated. Data shown were pooled from three independent experiments

We next assessed whether MDR *P. aeruginosa* infection of hma mice with induced acute ileitis was accompanied by distinct changes in intestinal microbiota composition. To address this, mice were sacrificed at day 7 following ileitis induction and cultural as well as culture-independent (i.e. molecular) analyses of intestinal luminal contents performed. Cultural analyses revealed total bacterial counts of up to 10^11^ CFU per g feces and increased loads of Gram-negative bacterial groups including enterobacteria and obligate anaerobic *Bacteroides/Prevotella* spp. (approximately 10^10^ CFU/g) as well as of enterococci (approximately 10^9^–10^10^ CFU/g) and obligate anaerobic *Clostridium/Eubacterium* spp. (approximately 10^9^–10^10^ CFU/g) in ileal and colonic luminal contents of mice suffering from acute ileitis (Fig. 3a). Aerobic Gram-positive rods, however, were virtually absent in small and large intestines of *T. gondii* infected mice (Fig. 3a). Notably, loads of the main cultivable bacterial groups did not differ between *P. aeruginosa* infected and uninfected mice with acute ileitis (Fig. 3a). In order to assess fastidious and non-cultivable bacteria we additionally performed quantitative molecular (i.e. 16S rRNA based) analyses of ileal contents. Interestingly, bifidobacterial gene numbers were more than two orders of magnitude lower in *P. aeruginosa* infected as compared to uninfected mice with acute ileitis (p < 0.05; Fig. 3b). The remaining bacterial groups, however, did not differ between the ilea of both cohorts (Fig. 3b), thus confirming results obtained from culture.

### Clinical, macroscopic and microscopic sequelae of multidrug resistant *P. aeruginosa* infection of human microbiota-associated mice suffering from acute ileitis

**Fig. 4:**
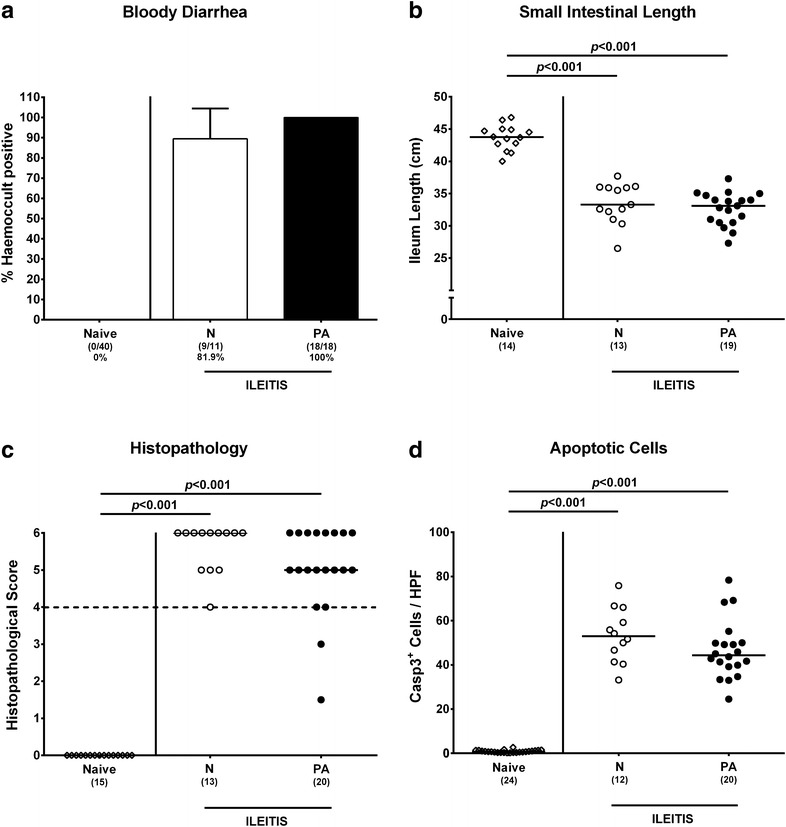
Clinical, macroscopic and microscopic sequelae in multidrug resistant *P. aeruginosa* infected human microbiota-associated mice suffering from acute ileitis. Human microbiota-associated mice were perorally challenged with *T. gondii* ME49 to induce acute ileitis and either additionally infected with MDR *P. aeruginosa* 3 days following ileitis induction (PA; *filled bar or circles*) or not (N; *open bar or circles*). Naive mice served as negative controls (*open diamonds*). Clinical, macroscopic and microscopic intestinal changes were assessed at day 7 post ileitis induction: **a** Abundance of bloody diarrhea was determined in fecal samples by the Guajac (Haemoccult) method (in %). Means, standard deviations and numbers of fecal blood positive mice out of the total numbers of animals (in *parentheses*) are indicated. **b** Absolute small intestinal lengths were measured (in cm) and **c** histopathological changes were determined in H&E stained ileal paraffin sections applying a standardized scoring system. Scores ≥4 (*dotted line*) indicate severe inflammation (with necrosis). **d** The average numbers of apoptotic cells (positive for caspase 3; Casp3^+^) from at least six high power fields (HPF, ×400 magnification) per animal were determined microscopically in immunohistochemically stained ileal paraffin sections. Numbers of animals (in *parentheses*), medians and significance levels (*p* values) determined by Kruskal–Wallis test or ordinary one-way ANOVA are indicated. Data shown were pooled from three independent experiments

We next investigated whether the clinical outcome of hma mice with acute ileitis was further deteriorated by MDR *P. aeruginosa* infection. Seven days post ileitis induction, blood could be detected microscopically or even macroscopically in fecal samples from any of *P. aeruginosa* infected hma mice, but only in 81.9% of hma mice without concomitant infection, whereas naive hma control animals were unaffected (Fig. 4a). Given that acute intestinal inflammation is associated with significant shortening of the inflamed gut [11, 16], we measured the small intestinal lengths of mice at necropsy. Both *P. aeruginosa* infected and uninfected hma mice with ileitis displayed comparably shorter small intestines than naive controls (p < 0.001; Fig. 4b). The comparable macroscopic sequelae of *P. aeruginosa* infected and uninfected hma mice with acute ileitis were paralleled by similar microscopic intestinal inflammatory changes. At day 7 post ileitis induction hma mice displayed destruction of villous architecture, cellular shedding into the lumen and massive necrosis of the ileal mucosa, irrespective of *P. aeruginosa* infection, as indicated by similarly elevated histopathological scores (p < 0.001 vs naive; Fig. 4c). In addition, ileal epithelial apoptotic cell numbers were comparably increased in *P. aeruginosa* infected and uninfected mice 7 days following ileitis induction (p < 0.001 vs naive; Fig. 4d). Taken together, *T. gondii* induced clinical, macroscopic and microscopic small intestinal changes did not further worsen upon MDR *P. aeruginosa* infection.

### Intestinal pro-inflammatory immune responses in multidrug resistant *P. aeruginosa* infected human microbiota-associated mice with acute ileitis

**Fig. 5:**
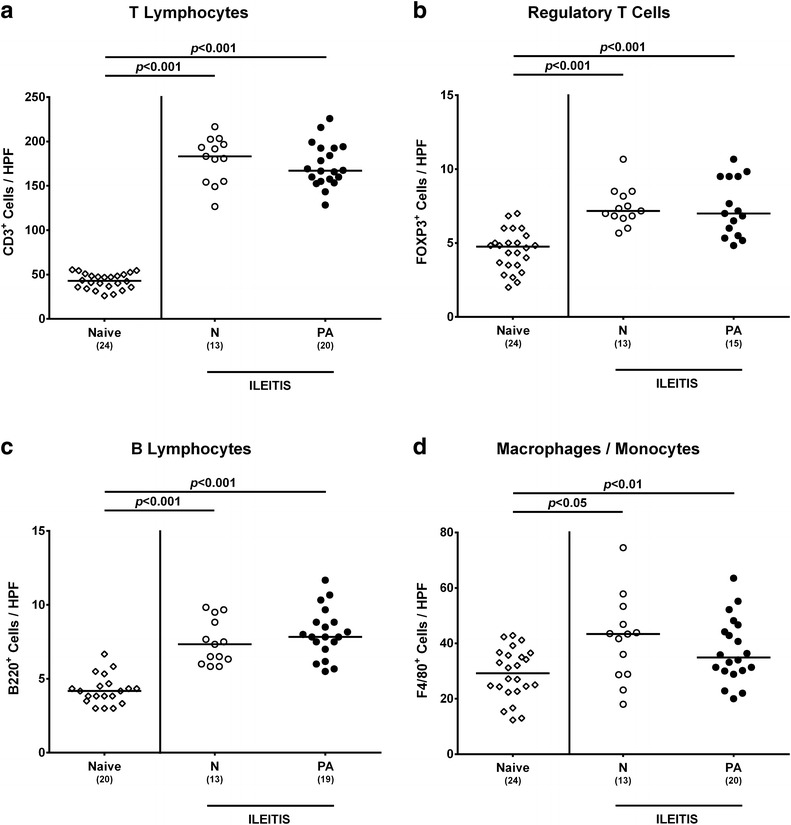
Small intestinal immune cell responses in multidrug resistant *P. aeruginosa* infected human microbiota-associated mice suffering from acute ileitis. Human microbiota-associated mice were perorally challenged with *T. gondii* ME49 to induce acute ileitis and either additionally infected with MDR *P. aeruginosa* 3 days following ileitis induction (PA; *filled circles*) or not (N; *open circles*). The average number of ileal **a** T lymphocytes (positive for CD3), **b** regulatory T cells (positive for FOXP3), **c** B lymphocytes (positive for B220), and **d** macrophages and monocytes (positive for F4/80) from six representative high power fields (HPF, ×400 magnification) per animal was determined microscopically in immunohistochemically stained ileal paraffin sections at day 7 post ileitis induction. Naive mice served as negative controls (*open diamonds*). Numbers of mice (in *parentheses*), medians (*black bars*) and significance levels (*p* values) determined by Kruskal–Wallis test or ordinary one-way ANOVA are indicated. Data shown were pooled from three independent experiments

**Fig. 6:**
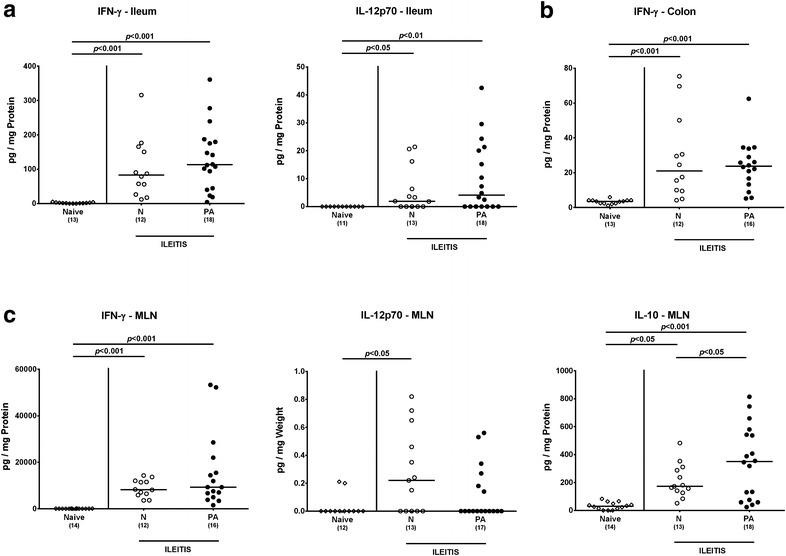
Intestinal cytokine responses in multidrug resistant *P. aeruginosa* infected human microbiota-associated mice suffering from acute ileitis. Human microbiota-associated mice were perorally challenged with *T. gondii* ME49 to induce acute ileitis and either additionally infected with MDR *P. aeruginosa* 3 days following ileitis induction (PA; *filled circles*) or not (N; *open circles*). At day 7 post ileitis induction secretion of distinct pro- and anti-inflammatory cytokines were determined in ex vivo biopsies derived from different intestinal compartments including **a** ileum (IFN-γ and IL-12p70), **b** colon (IFN-γ) and **c** MLN (IFN-γ, IL-12p70, IL-10). Naive mice served as negative controls (*open diamonds*). Numbers of mice (in *parentheses*), medians (*black bars*) and significance levels (*p* values) determined by Kruskal–Wallis test or ordinary one-way ANOVA are indicated. Data shown were pooled from three independent experiments

We next surveyed quantitative small intestinal immune cell responses upon MDR *P. aeruginosa* infection of hma mice suffering from acute ileitis applying in situ immunohistochemistry. At day 7 post ileitis induction, both *P. aeruginosa* infected and uninfected hma mice displayed multifold and comparably elevated numbers of T and B lymphocytes, Treg as well as of macrophages and monocytes within the ileal mucosa and lamina propria (Fig. 5). Hence, *T. gondii* infection resulted in a profound increase of innate and adaptive immune cells in the distal small intestines that was, however, not further enhanced by MDR *P. aeruginosa* infection. This was also true for intestinal pro-inflammatory cytokine levels as indicated by similarly elevated IFN-γ concentrations in ex vivo biopsies derived from ileum and MLN (p < 0.001) and additionally of IL-12p70 (p < 0.05 and p < 0.01, respectively) in the ileum of *P. aeruginosa* infected and uninfected hma mice 7 days following *T. gondii* infection (Fig. 6a, c). Furthermore, IL-12p70 levels were increased in the MLN of uninfected, but not *P. aeruginosa* infected hma mice with ileitis induction (p < 0.05; Fig. 6c). Interestingly, the anti-inflammatory cytokine IL-10 increased multifold upon ileitis induction in either mice (p < 0.05 and p < 0.001, respectively), but more distinctly in *P. aeruginosa* infected as compared to uninfected hma mice (p < 0.05; Fig. 6c). Given that upon peroral *T. gondii* infection the small, but not large intestinal tract has been described as exclusive intestinal predilection site of inflammation so far [13], we unexpectedly observed elevated IFN-γ secretion even in supernatants of colonic ex vivo biopsies at day 7 post ileitis induction in either mice (p < 0.001; Fig. 6b).

### Extra-intestinal pro-inflammatory immune responses in multidrug resistant *P. aeruginosa* infected human microbiota-associated mice with acute ileitis

**Fig. 7:**
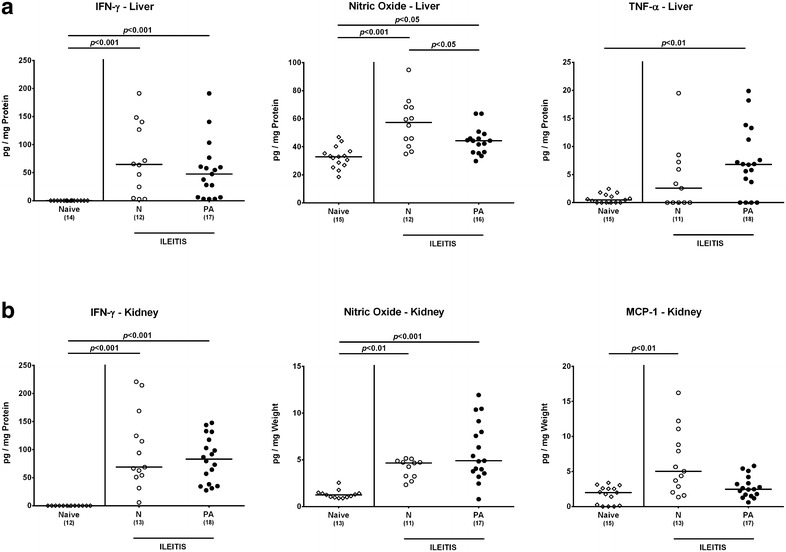
Extra-intestinal cytokine responses in multidrug resistant *P. aeruginosa* infected human microbiota-associated mice suffering from acute ileitis. Human microbiota-associated mice were perorally challenged with *T. gondii* ME49 to induce acute ileitis and either additionally infected with MDR *P. aeruginosa* 3 days following ileitis induction (PA; *filled circles*) or not (N; *open circles*). At day 7 post ileitis induction secretion of distinct pro-inflammatory cytokines were determined in ex vivo biopsies derived from different extra-intestinal compartments including **a** liver (IFN-γ, nitric oxide, TNF-α) and **b** kidney (IFN-γ, nitric oxide, MCP-1). Naive mice served as negative controls (*open diamonds*). Numbers of mice (in *parentheses*), medians (*black bars*) and significance levels (*p* values) determined by Kruskal–Wallis test or ordinary one-way ANOVA are indicated. Data shown were pooled from three independent experiments

We next addressed whether MDR *P. aeruginosa* infection worsened potential extra-intestinal inflammatory responses in hma mice with acute ileitis. Interestingly, IFN-γ and nitric oxide secretion increased in ex vivo biopsies taken from liver and kidney until day 7 following ileitis induction in both *P. aeruginosa* infected and uninfected hma mice (p < 0.05 to 0.001; Fig. 7), whereas nitric oxide levels were slightly lower in the liver of the latter as compared to the former (p < 0.05; Fig. 7a). Notably, hepatic TNF-α concentrations were elevated in *P. aeruginosa* infected hma mice only (p < 0.01; Fig. 7a), but renal MCP-1 levels increased exclusively in uninfected hma mice with ileitis (p < 0.01; Fig. 7b). Hence, ileitis induction is in fact accompanied by pro-inflammatory responses in extra-intestinal compartments. However, except for hepatic TNF-α levels MDR *P. aeruginosa* infection does not result in more distinct pro-inflammatory cytokine responses in liver and kidney of hma mice with acute ileitis.

### Systemic sequelae of multidrug resistant *P. aeruginosa* infection in human microbiota-associated mice with acute ileitis

**Fig. 8:**
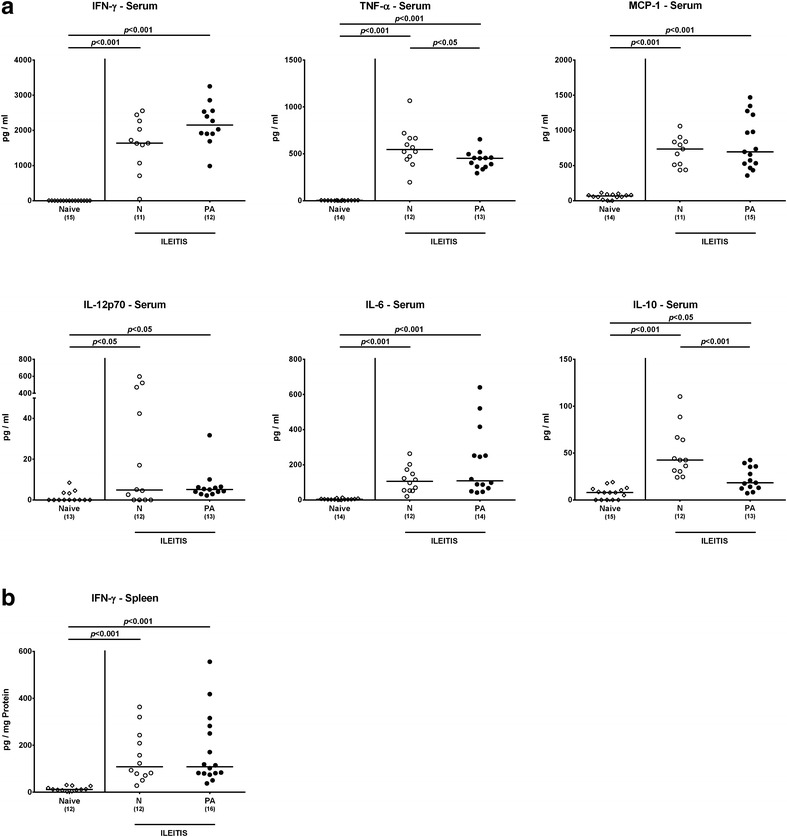
Systemic cytokine responses in multidrug resistant *P. aeruginosa* infected human microbiota-associated mice suffering from acute ileitis. Human microbiota-associated mice were perorally challenged with *T. gondii* ME49 to induce acute ileitis and either additionally infected with MDR *P. aeruginosa* 3 days following ileitis induction (PA; *filled circles*) or not (N; *open circles*). At day 7 post ileitis induction secretion of distinct pro- and anti-inflammatory cytokines were determined in systemic compartments such as **a** serum (IFN-γ, TNF-α, MCP-1, IL-12p70, IL-6 and IL-10) and **b** spleen (IFN-γ). Naive mice served as negative controls (*open diamonds*). Numbers of mice (in *parentheses*), medians (*black bars*) and significance levels (*p* values) determined by Kruskal–Wallis test or ordinary one-way ANOVA are indicated. Data shown were pooled from three independent experiments

We further investigated whether MDR *P. aeruginosa* infection aggravated potential systemic responses upon ileitis induction. At day 7 following *T. gondii* infection, pro-inflammatory cytokines including IFN-γ, TNF-α, MCP-1, IL-12p70 and IL-6 were elevated in both *P. aeruginosa* infected and uninfected hma mice with ileitis as compared to naive controls (p < 0.05-0.001; Fig. 8a) that were accompanied by increased IFN-γ secretion in splenic ex vivo biopsies (p < 0.001; Fig. 8b). Upon ileitis induction, serum IL-10 concentrations increased in either hma mice, but less distinctly in *P. aeruginosa* infected mice as compared to uninfected controls (p < 0.001; Fig. 8a). Hence, MDR *P. aeruginosa* infection did not exacerbate systemic pro-inflammatory sequelae of acute ileitis, but resulted in lower anti-inflammatory responses.

### Bacterial translocation following multidrug resistant *P. aeruginosa* infection of human microbiota-associated mice with acute ileitis

**Fig. 9:**
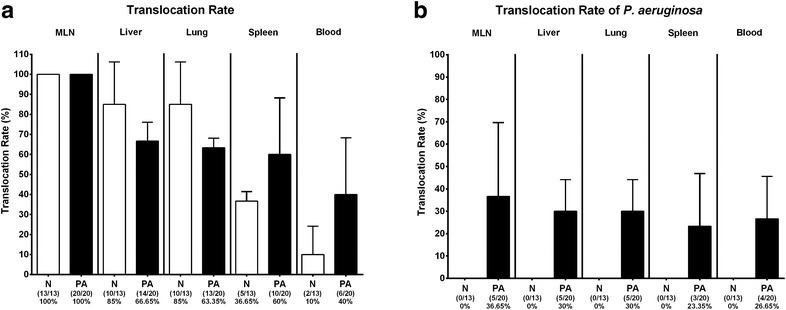
Translocating intestinal bacteria in multidrug resistant *P. aeruginosa* infected human microbiota-associated mice suffering from acute ileitis. Human microbiota-associated mice were perorally challenged with *T. gondii* ME49 to induce acute ileitis and either additionally infected with MDR *P. aeruginosa* 3 days following ileitis induction (PA; *filled bars*) or not (N; *open bars*). At day 7 post ileitis induction translocation rates of **a** intestinal bacterial species and of **b** *P. aeruginosa* to extra-intestinal and systemic compartments were determined by cultivation of homogenated ex vivo biopsies derived from mesenteric lymph nodes (MLN), liver, lung and spleen (direct plating on solid media) and of cardiac blood (in thioglycolate enrichment broths) with subsequent subcultivation and species identification. Mean translocation rates (%) ±SD and absolute numbers of positive samples out of total number analyzed (in *parentheses*) are indicated. Data shown were pooled from three independent experiments

We next analyzed whether viable bacteria originating from the intestinal microbiota were abundant in extra-intestinal and systemic compartments of hma mice suffering from acute ileitis, and whether MDR *P. aeruginosa* infection facilitated bacterial translocation. In all MLN derived from *P. aeruginosa* infected and uninfected hma mice with ileitis, commensal intestinal bacteria such as enterobacteria, enterococci, lactobacilli, *Bacteroides/Prevotella* spp. and/or *Clostridium/Eubacterium* spp. could be cultured. In extra-intestinal compartments such as liver, however, mean bacterial translocation rates were 66.7% ± 9.4 and 85.0% ± 21.2 in *P. aeruginosa* infected and uninfected mice with ileitis, respectively, whereas commensal intestinal species could be cultured in 63.4% ± 4.8 and 85.0% ± 21.2 of lungs in respective cohorts (Fig. 9a). Interestingly, viable intestinal bacteria were more frequently detected in systemic compartments such as spleen and cardiac blood of *P. aeruginosa* infected as compared to uninfected mice at day 7 following ileitis induction (mean translocation rates spleen: 60.0% ± 28.3 vs 36.7% ± 4.7, respectively; cardiac blood: 40.0% ± 28.3 vs 10.0% ± 14.1, respectively) (Fig. 9a). *P. aeruginosa* could be isolated on average from approximately 25–40% of respective organ homogenates and blood (Fig. 9b; Additional file 1: Figure S1). Notably, no translocating intestinal bacterial species could be cultured from respective extra-intestinal and systemic compartments of naive hma mice (not shown).

## Discussion

In the present study we were able to show that an acute intestinal inflammatory condition facilitates MDR *P. aeruginosa* infection of the vertebrate host. Whereas (already fatal) ileal inflammatory changes were similar, extra-intestinal sequelae of high dose *T. gondii* infection were further aggravated by subsequent *P. aeruginosa* infection.

Applying a well-known infection model of acute *T. gondii* induced ileitis in (with respect to their microbiota) “humanized” mice, we first verified that prior to ileitis induction, human microbiota had stably established within the murine host and that microbiota composition was comparable in experimental groups that were subsequently infected with MDR *P. aeruginosa* or remained uninfected. These results are in accordance with our previous studies, where gnotobiotic (i.e. secondary abiotic) mice were replenished with human microbiota in order to overcome colonization resistance against *Campylobacter jejuni*. Quantitative molecular analysis further revealed that following peroral fecal transplantation human microbiota could stably establish within the intestinal tract for more than 6 weeks [16]. Moreover, we showed previously that following peroral infection with high doses (i.e. >50 cysts) of *T. gondii* mice developed a distinct Th1-type immunopathology and exhibited an overgrowth of the inflamed ileal lumen with commensal Gram-negative bacterial species such as *Escherichia coli* (*E. coli*) and *Bacteroides/Prevotella* spp. [11]. Due to progressive virulence of the applied *T. gondii* ME49 strain by repeated passages in NMRI “bank” mice for several generations, we also subjected hma mice to 50 cysts in the present study instead of 100 cysts as in our previous reports [10–12, 14, 22, 24–29].

Remarkably, acute ileitis induced by 50 cysts of *T. gondii* rendered hma mice susceptible to MDR *P. aeruginosa* infection. This is in accordance with our previous studies demonstrating that hma mice suffering from small intestinal inflammation could be stably colonized by *C. jejuni* [27]. It is hence tempting to speculate that inflammation induced changes of the intraluminal milieu within the intestinal tract predisposes the host for infection with obligate enteropathogenic species such as *C. jejuni*, but also opportunistic pathogens including *P. aeruginosa*. Our hypothesis is further supported by a study showing that a nonpathogenic intestinal *E. coli* isolate as well as the pathogen *Salmonella typhimurium* were able to outcompete the endogenous microbiota of IL-10^−/−^ mice, a murine model of chronic colitis, suggesting that growth of Gram-negative enterobacteria is enhanced by host mediated intestinal inflammation and following modification of the composition of intestinal microbiota [30, 31]. Furthermore, in a murine co-infection model intestinal colonization capacity of *S. typhimurium* was enhanced by inflammatory changes in the intestinal mucosa and dysbiosis elicited by *Plasmodium yoelii* infection [32].

Given that the intestinal intraluminal milieu is determined by a plethora of factors including the bacteria residing in the intestinal compartments, we performed a comprehensive quantitative survey of the intestinal microbiota composition. Cultural and molecular analyses of the predominant intestinal bacterial groups revealed that differences in the overall microbiota composition of *P. aeruginosa* infected and uninfected hma mice with induced ileitis were rather subtle at the first glance. Ileal loads of bifidobacteria, however, were more than two orders of magnitude lower following *P. aeruginosa* infection. As commensal residents with beneficial effects for intestinal homeostasis, bifidobacteria contribute to mucosal barrier functions directed against pathogenic colonization of the vertebrate host [33, 34]. Two bifidobacterial strains that were isolated from resident infant human gastrointestinal microbiota exerted antibacterial activity against several pathogens such as *S. typhimurium, E. coli, Klebsiella pneumoniae, Yersinia pseudotuberculosis, Staphylococcus aureus* and *P. aeruginosa* in vitro [34]. In addition, in vitro as well as in vivo studies revealed that *Bifidobacterium animalis* AHC7, for instance, exerts an anti-inflammatory effect through the attenuation of NF-κB activation in response to murine *S. typhimurium* infection and that stimulation of dendritic cells with *B. animalis* AHC7 significantly increased CD25+Foxp3+ T cell (Treg) numbers [35]. We could further show previously that conventionally colonized mice deficient in the innate immune-receptor nucleotide-oligomerization-domaine-2 (NOD2) were virtually lacking the bifidobacterial population in their intestines and (at least in part) exhibited compromised host resistance, reduced local anti-inflammatory and increased systemic pro-inflammatory immune response upon peroral high dose *T. gondii* infection [29].

Given that acute *T. gondii* induced ileitis is highly dependent on Toll-like Receptor (TLR)-4 mediated signaling of lipopolysaccharide derived from Gram-negative intestinal commensals [11, 15], one could speculate that additional infection with a Gram-negative bacterium such as *P. aeruginosa* might further exacerbate the induced inflammatory process. Our study, however, revealed that clinical and ileal changes, the profound influx of innate and adaptive immune cells into the distal small intestines and increased secretion of pro-inflammatory cytokines in ileum and MLN following *T. gondii* infection were not further aggravated by MDR *P. aeruginosa* infection. This is surprising considering the plethora of virulence factors of *P. aeruginosa*. For instance, *Pseudomonas* lipid A, a core component of bacterial lipopeptide, has been shown to activate NFκB signaling through TLR-4 and subsequent pro-inflammatory cytokine secretion [36]. In turn, neutrophils are recruited to the infection site and contribute to the inflammatory host response to *P. aeruginosa* [1]. One needs to take into consideration, however, that peroral high dose *T. gondii* infection (irrespective whether performed with 50 or 100 cysts) results in a profound Th1-driven hyper-inflammatory scenario (“cytokine tsunami”) [13] that cannot further be deteriorated by additional MDR *P. aeruginosa* infection. Strikingly, we were able to observe elevated IFN-γ secretion not only in ileal, but also colonic ex vivo biopsies 7 days post ileitis induction. To our best knowledge, the terminal ileum has been reported as exclusive predilection site following peroral high dose *T. gondii* infection of conventionally colonized mice so far [13].

Remarkably, extra-intestinal sequelae of acute ileitis induction were further amplified by *P. aeruginosa* challenge as indicated by even more pronounced increases in hepatic TNF-α concentrations following *P. aeruginosa* infection of *T. gondii* pre-infected mice. Potential inflammatory effects of MDR *P. aeruginosa* infection in the small intestines (and beyond) should therefore be more distinctly deciphered either in a less acute (i.e. less severe) or more chronic infection model following peroral low dose infection with less than 10 cysts of *T. gondii*, for instance. To the best of our knowledge, however, a chronic *T. gondii* ileitis model has not been established so far. Alternatively, experimental models of large intestinal inflammation could be applied to investigate host susceptibility and the pro-inflammatory potential of peroral MDR *P. aeruginosa* infection during intestinal inflammation in a better discriminatory way than with the model used here. To address this, the murine dextran sulfate sodium induced colitis model would be a promising candidate, for instance.

In the present study we were further able to demonstrate that anti-inflammatory IL-10 levels were multifold increased in the MLN following ileitis induction, but even more distinctly upon additional *P. aeruginosa* infection, whereas conversely, serum IL-10 concentrations were lower in *P. aeruginosa* infected as compared to non-infected hma mice suffering from acute ileitis. Hence, the more pronounced intestinal anti-inflammatory response upon *P. aeruginosa* application was not sufficient to counteract the pro-inflammatory sequelae caused by *T. gondii* and *P. aeruginosa* co-infection. One might have also expected comparable increases in systemic IL-10 levels, given that translocation of viable bacteria originating from the intestinal microbiota to systemic compartments such as spleen and blood had occurred more frequently in *P. aeruginosa* co-infected versus non-infected hma mice with acute ileitis.

We were finally able to show that viable intestinal bacteria were more frequently detected in systemic compartments such as spleen and blood of *P. aeruginosa* infected as compared to uninfected hma mice with induced ileitis indicating that (even though not otherwise determined in our study) epithelial barrier leakage was supposably even more pronounced by *P. aeruginosa* co-challenge than by acute ileitis induction alone, further facilitating bacterial translocation to extra-intestinal including systemic sites. Notably, several studies illustrate a link between the etiology of inflammatory bowel diseases (IBD) including Crohn’s disease and the abundance of *Pseudomonas* species such as *P. fluorescens* within the intestinal mucosa [37–39]. Solid data regarding the role of *P. aeruginosa* in IBD pathogenesis, however, are scarce. One study applying molecular analysis of *Pseudomonas* specific 16S RNA in ileal tissue samples derived from children suffering from Crohn’s disease revealed a higher prevalence of several *Pseudomonas* species including *P. proteolytica* and *P. brenneri* in pediatric Crohn’s disease patients as compared to control individuals without IBD, whereas interestingly *P. aeruginosa* could be detected in non-IBD patients only [40]. In patients suffering from irritable bowel disease, however, detection rates of *P. aeruginosa* specific 16S RNA were increased in duodenal mucosa-associated biopsies and fecal samples [8]. Furthermore, a case report of a child suffering from ulcerative colitis revealed that *P. aeruginosa* 16S rRNA could be identified in colonic biopsies [9]. Already an older study from 1966 suggested a temporal correlation between identical *P. aeruginosa* strains isolated from patients’ lesions and feces pointing towards a spread of viable *P. aeruginosa* via the blood stream [7]. Meanwhile, there is evidence that the risk of developing a clinically manifest *P. aeruginosa* infection is significantly higher upon rectal colonization of patients in intensive care units [41].

## Conclusion

Taken together, acute small intestinal inflammation renders hma mice susceptible to infection with MDR *P. aeruginosa*, but *P. aeruginosa* infection does neither further deteriorate ileal, nor extra-intestinal sequelae of ileitis induction. We conclude that intestinal inflammation might also pose particularly hospitalized patients at risk for acquisition of MDR Gram-negative bacteria including *P. aeruginosa*. Given the importance of the interaction between *P. aeruginosa*, the intestinal microbiota and the host immune system as shown here, future studies applying less acute and/or more chronic in vivo infection models are needed for a better understanding of the underlying molecular mechanisms.

## Additional file

**Additional file 1: Figure S1.** Translocating multidrug resistant *P. aeruginosa* in infected human microbiota-associated mice suffering from acute ileitis. Human microbiota-associated mice were perorally challenged with *T. gondii* ME49 to induce acute ileitis and either additionally infected with MDR *P. aeruginosa* 3 days following ileitis induction (PA; filled bars) or not (N; open bars). At day 7 following ileitis induction *P. aeruginosa* loads were quantitatively assessed in extra-intestinal and systemic compartments such as mesenteric lymph nodes (MLN), liver, lung, spleen and cardiac blood by direct plating. Absolute numbers of positive samples out of total number analyzed are indicated in parentheses. Data shown were pooled from three independent experiments.

## Abbreviations

CFU: colony forming units
*E. coli*: *Escherichia coli*
hma: human microbiota-associated
IBD: inflammatory bowel diseases
IFN: interferon
IL: interleukin
MCP: monocyte chemoattractant protein
MLN: mesenteric lymph nodes
MDR: multidrug resistant
*P. aeruginosa*: *Pseudomonas aeruginosa*
PBS: phosphate buffered saline
p.i.: post infection
qRT-PCR: quantitative real-time polymerase chain reaction
*T. gondii*: *Toxoplasma gondii*
Th1: T helper cell 1
TNF-α: tumor necrosis factor-α
TLR: toll-like receptor
Treg: regulatory T cells
