# Supplementary figures and images for: Acute ileitis facilitates infection with multidrug resistant Pseudomonas aeruginosa in human microbiota-associated mice
Source: Gut Pathog. 2017 Jan 18;9:4. doi: 10.1186/s13099-017-0154-4 (PMC5241993; doi:10.1186/s13099-017-0154-4)

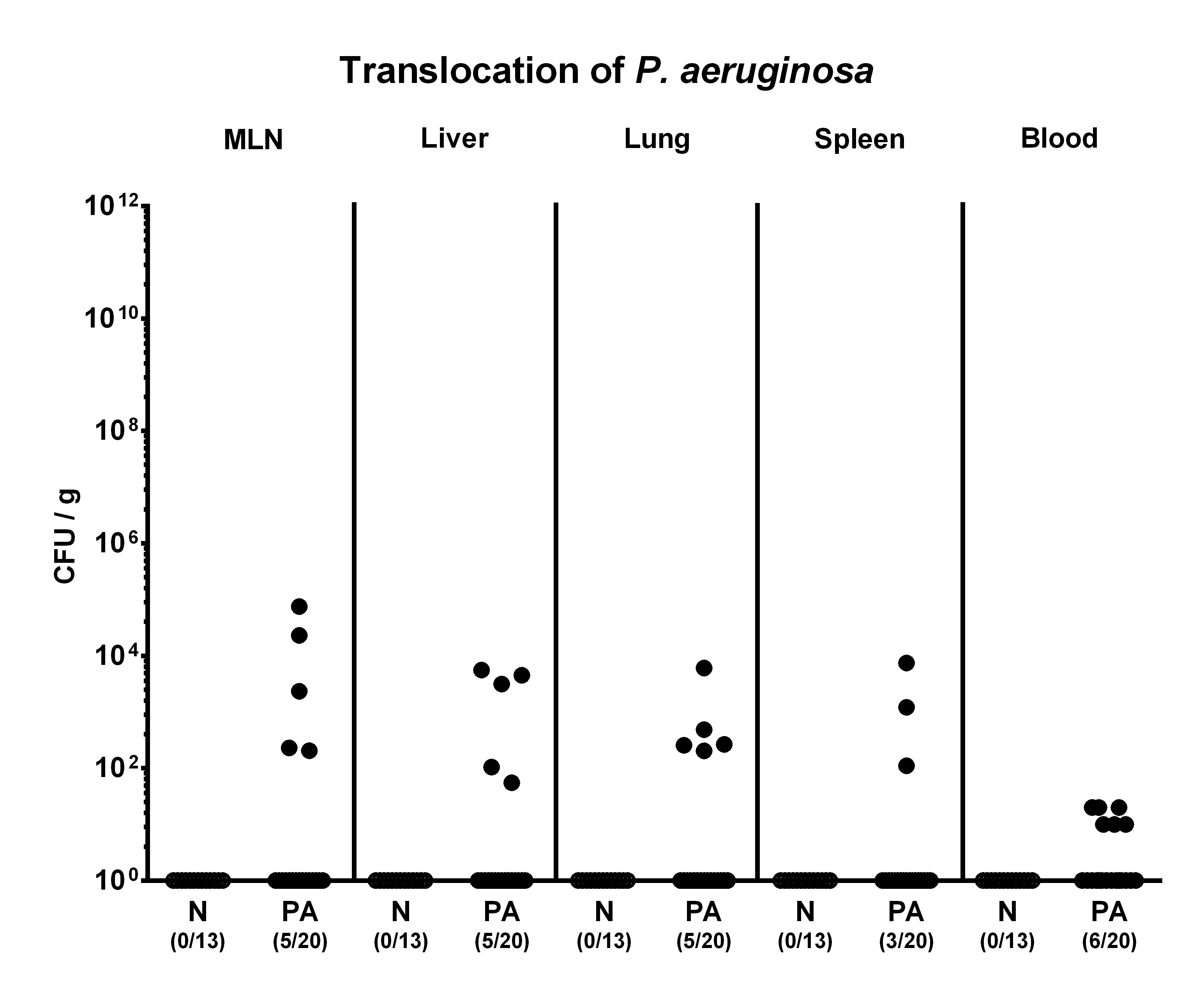

Supplement: Supplementary file 1 — Additional file 1: Figure S1. Translocating multidrug resistant P. aeruginosa in infected human microbiota-associated mice suffering from acute ileitis. Human microbiota-associated mice were perorally challenged with T. gondii ME49 to induce acute ileitis and either additionally infected with MDR P. aeruginosa 3 days following ileitis induction (PA; filled bars) or not (N; open bars). At day 7 following ileitis induction P. aeruginosa loads were quantitatively assessed in extra-intestinal and systemic compartments such as mesenteric lymph nodes (MLN), liver, lung, spleen and cardiac blood by direct plating. Absolute numbers of positive samples out of total number analyzed are indicated in parentheses. Data shown were pooled from three independent experiments. [file 13099_2017_154_MOESM1_ESM.tiff]
